# The Influence of Household and Community Food Environments on Food Insecurity in Limpopo Province, South Africa

**DOI:** 10.3390/ijerph21020125

**Published:** 2024-01-24

**Authors:** Xikombiso Gertrude Mbhenyane, Ayuk Betrand Tambe

**Affiliations:** Division of Human Nutrition, Faculty of Medicine and Health Sciences, Stellenbosch University, Cape Town 8000, South Africa

**Keywords:** household, hunger, community food environments, food insecurity, rural setting, South Africa

## Abstract

Insufficient access to enough quality food remains a public health challenge in South Africa. This study aimed to report the influence of community and the household environment, food system inventories, and food procurement on household food security. The findings alluded that food insecurity is prevalent among the rural areas in South Africa. Large household sizes and a limited access to and use of water and food storage facilities for food preservation are the major household determinants. A multi-sectoral nutrition-planning approach that accelerates the achievement of the Sustainable Development Goals should be reinforced. We recommend the promotion of food production for consumption in rural communities.

## 1. Introduction

The terms “food security” and “insecurity” have been used to define whether or not households have access to a sufficient quality and quantity of food. Insufficient access to quality food remains a public health challenge, as it affected about 821 million people globally in 2017 [1]. Regardless of the outstanding declines in the global rates of food insecurity since 2000, the levels of food insecurity in Asia and Africa are still considered serious or disturbing according to the Global Hunger Index (GHI) [2]. Domestic food price inflation remains high, especially in South Asia and Africa, according to the World Bank in 2023 [3]. Evidence from 2017 confirms that lower levels of per capita food consumption in some countries in Asia and Africa have contributed significantly to insufficient dietary energy consumption in these countries [1]. Therefore, the state of global food security does not guarantee food security at the national level in different countries.

Achieving Sustainable Development Goals (SDGs) two and three of a world without hunger and any form of malnutrition by 2030 remains a challenge in the face of these signs of increasing food insecurity and malnutrition [4]. What, then, can be done to hasten progress given that studies have shown that more than enough food is produced by farmers around the globe to feed its ten billion people and even more [5]? Thus, there is a need to investigate the determinants of household hunger other than food production that are insufficient to effectively address the issues of hunger and malnutrition.

South Africa is considered an upper-middle-income country according to the World Bank classification [6], and produces enough food to feed its population. However, studies have shown that the country is characterized by large income inequality and poverty, with a bulk number of households within the country still food-insecure [7]. It has been reported that food insecurity and inadequate nutrition are prevalent among the rural and urban poor areas of South Africa as a result of unemployment [8].

Many households in South Africa receive social welfare grants, and beneficiaries are estimated at 46% of the population. This means that approximately 27.8 million South Africans are social grant recipients [9]. Social welfare grants are financial assistance programs provided by the government to individuals and families who need support. These grants aim to alleviate poverty, reduce inequality, and improve the overall well-being of vulnerable populations [10]. There are five major social security grants in South Africa: the State Old Age Pension, the Disability Grant, the Child Support Grant, the Foster Child Grant, and the Care Dependency Grant [9]. This adds up to more than 40% of the 62-million population [11]. In 2019, the number of people receiving social grants in Limpopo province was 2,312,000 [12] of the estimated 5,799,090 population [13].

Furthermore, the rise in the cost of food and the economy’s consumer inflation level is another factor contributing to food insecurity, as the population is forced to change their eating habits by adopting short-term strategies [14]. Zimmer et al. [15] also asserted that food price shocks driven by climate variability and household-scale economic shocks caused by job losses or unexpected expenses may affect the ability of households to purchase food, causing them to use alternative coping strategies such as engaging in food production in their backyard or food sharing among neighbors.

Reardon et al. [16] reported the shift from home processing, for example, hand-pounding grain, to purchasing low-processed products like milled grain and oil, and highly processed products, like traditional fritters, and then packaged foods, like cookies and bread. Zimmer et al. [15] also reported that rural people now depend on food purchases, with rural sources, on average, slightly further away (>40 km) than urban sources. Yet, in the context of the South African poor, the social grant does not provide sufficient resources for food acquisition. The agricultural producer support and development program in South Africa has a sub-program for food security. This sub-program coordinates and implements various food production initiatives as highlighted and adopted in the National Food and Nutrition Security Policy for South Africa. It also provides information and facilitates training for community projects and households benefiting from these food security initiatives.

To understand the state of household food security, it is necessary to assess the household biophysical environment, food systems, and food procurement trends in rural South Africa and identify potential pathways for intervention. A food system is defined as a set of activities and processes, ranging from food production to the consumption of food, which involves interactions between people and the environment that affect food security outcomes [17,18], whereas food procurement is about the process of how food is purchased and used within a household. According to Rideout, Mah, and Minaker [19], food outlets, or the density or variety of diverse types of food outlets within a specific geographic area, constitute community food environments.

Mazenda et al. [20] studied household-level determinants of food security in the City of Tshwane, South Africa. Their results indicated that a younger age, gender (male), lower education, unemployment, a large household size, low income, and grant type were positively significant at the 1% level regarding extreme changes in food insecurity. Only the grant was insignificant regarding mild changes in food security. The study concluded that there are extremes of food insecurity and severe food insecurity indicating inequality, with other various socioeconomic contributors. The Auditor-General of South Africa’s [21] First Special Report in 2020 on the Financial Management of the Government’s COVID-19 initiatives has revealed that female-headed households continue to endure the most food insecurity and nutrition-related challenges. In addition, households that have a high number of adult females and young children live in conditions that are plagued by exacerbated levels of poverty. There is a need to examine the household and community food environments’ influence on food insecurity in rural communities.

The extreme weather events that are being experienced in South Africa, such as floods and drought, are becoming more intense due to the growing climate crisis. Other disasters that South Africa has experienced include tornados, flash floods, droughts, strong winds, severe thunderstorms, sudden cold spells, and infected water supplies. Such disruptions can generate a period of transitory or episodic food insecurity, where people’s access to adequate food that supports their well-being is hindered [22]. Communities often become homeless and internal migration occurs, which has the potential to impact food security [22]. During these periods, often unexpected or seasonal, people are internally displaced and lack or have difficulty accessing nutritious and culturally appropriate foods. The duration of these effects on people’s food security may vary due to the magnitude of the impact, coupled with an affected population’s vulnerability stemming from social–ecological characteristics, such as race, sex, geography, economics, politics, ecosystem services, and the biophysical aspects of a place, among others. Social–ecological characteristics may predispose some populations to be at higher risk of harm and may reduce their ability to recover from disaster impacts.

Mkhize, Mthembu, and Napier [23] conducted a study titled “Transforming a local food system to address food and nutrition insecurity in an urban informal settlement area immediately after the KwaZulu-Natal 2022 floods”. Infrastructure was destroyed and internal migration ensued. They reported that there is a direct link between food and nutrition security and households in urban informal settlements, which can be associated with household economic status and food environments. Their recommendation included that communities must be actively encouraged to be agents of change, even in the face of disasters, by being educated regarding the importance of local food systems and how they can contribute to the food value chain for sustainable food security.

This research aims to investigate the influence of the household biophysical environment, food procurements, community food environments, and food systems on household food security.

## 2. Background

The South Africa Demographic and Health Survey [24] suggests that about 18% of adults either experienced or were at risk of hunger in 2016; the rates were 15% in urban areas, 27% in non-urban areas, and 33% and 3% in the lower and higher wealth quintiles, respectively. For children, the figures were 20% at the national level, 17% urban, 25% non-urban, and 28% and 6% for the lower and higher wealth quintiles, respectively. More recently, the onset of the COVID-19 pandemic in South Africa and the associated lockdowns brought nutrition, food security, and hunger into prominence, as crucial food access points were closed with limited transportation to marketplaces. The government came up with social relief programs that included food parcels and/or cash as protective measures against food insecurity. Non-governmental organizations, non-profit organizations, and individuals also set up feeding centers to respond to the impacts of the pandemic. The government implemented temporary emergency social support measures, which included (i) temporary “top-ups” to existing social grants, (ii) the establishment of a COVID-19 Social Relief Disaster grant, (iii) the introduction of the Temporary Employee/Employers Relief Scheme (TERS), and (iv) localized social relief [25,26,27]. The interventions were introduced to circumvent the possibility of food shortages in low-income households.

The High-Level Panel of Experts of the Committee on World Food Security of the Food and Agriculture Organization [28] asserts that the war between Russia and Ukraine has major implications for global food security and nutrition. The war has triggered new crises in food systems on top of existing challenges that were already undermining the global community’s goal of achieving Sustainable Development Goal #2: “Zero hunger”. Russia and Ukraine are both considered “global breadbaskets” and are important producers and exporters of vital agricultural commodities such as grains, minerals, fertilizers, and energy. Many researchers agree that the Russia–Ukraine war’s impact on food, fuel, and fertilizer prices is a major concern for global poverty and food insecurity [29,30,31,32,33,34,35,36]. In South Africa, there have been rising interest rates, rising inflation, and rising food and fuel prices. The cost of feeding a household with more than four members increased and resulted in the purchase of less preferred foods [20]. The consequences of this war and disruption in the food chain supply will have a long-term impact on nutrition and food security, especially in low- and middle-income countries. This will have an impact on Sustainable Development Goal #2: “Zero hunger”. Communities must devise methods to circumvent the threatening food shortages and high food prices. This is essential to ensure that food insecurity in vulnerable populations is kept low.

## 3. Methods

### 3.1. Study Design

This study employed a cross-sectional survey using quantitative techniques. The instruments used were a community asset inventory, including a market survey of food systems using an observation checklist. A household questionnaire that included sections on sociodemographic profiles and biophysical environmental profiles (including food availability/inventory), household food security [37], and coping strategies [38] was used. Individual anthropometric measurements, disease profiles, health risks, and clinical assessments were recorded in a form as part of the questionnaire. Dietary patterns and food consumption were assessed using 24 h recall and a food frequency questionnaire [39]. In addition, food taboos, food beliefs, and values were also determined.

### 3.2. Study Setting

**Figure 1:**
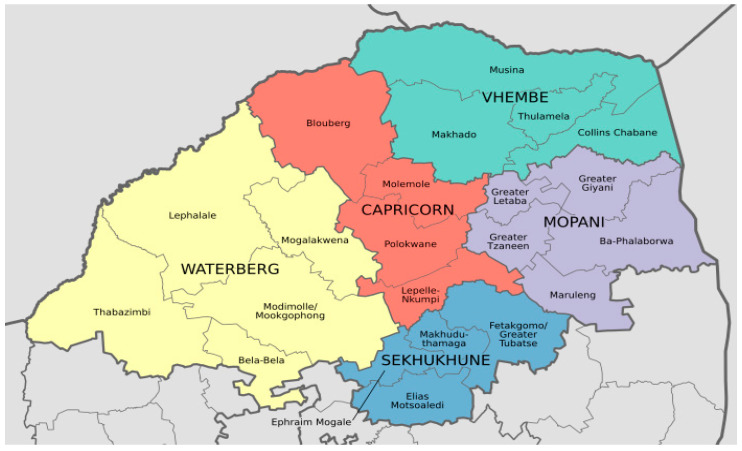
Map of Limpopo Province’s post-1994 demarcation of district municipalities. Source: researchgate.net. Available in the public domain via license CC BY 4.0.

The study was conducted in the Limpopo Province of South Africa. This northernmost province of South Africa shares international borders with three sub-Saharan countries: Botswana, Zimbabwe, and Mozambique. See Figure 1 below. The province is divided into five district municipalities divided into 25 local municipalities. The study took place in the two villages located in the Collins Chabane Local Municipality located in the Vhembe District bordering the Kruger National Park and Zimbabwe. See the map of Limpopo Province showing the local and district municipalities. These villages were selected because there were agricultural development projects. The Sweden African Development Fund funded a project in Village One to install sprinklers for irrigation for 15 subsistence farmers in 1994, whereas in Village Two, small-scale farmers benefited from agricultural schemes between 1985 and 1994 from the former homeland, which installed a water irrigation system and facilitated access to the market for their produce. There were 91 households in Village One and 645 households in Village Two [40] at the time of this study. The dependency ratio for Village One was 83.3%, and that of Village Two was 82.8% [40], signaling a low socioeconomic level.

### 3.3. Target Population

The target population was the households situated in the two selected villages. For Village One, all 91 households were sampled, while for Village Two, 200 of the 645 households were sampled using systematic sampling. The villages were purposively selected based on part of the multi-state sampling used to obtain the study areas. The reason for selecting these areas is due to a high level of food security, as reported in preceding studies [24], despite the food production support initiatives. All households in Village One were included since the total population was less than a hundred, while systematic sampling was used to select households in Village Two, with all households situated in the study areas having an equal chance of being selected

In Village Two, the village was divided into four sections, two from the old settlement and two from the new settlement. There were 645 households and 200 were sampled, which is 31% of the village population. A systematic random sample was followed, with a starting point selected upon arrival from a street, and every 5th household was selected. A household was defined as those living in the same yard and eating from the same pot. Furthermore, a “household” could be defined as an arrangement of co-residence with shared consumption and production. Families in the context of this study were either nuclear, extended, or complex, with two or more generations, grandparents, parents, children, and grandchildren. There were no joint families in these areas; however, siblings could live in the same household (often a parental home) with their children. Statistics South Africa [41] gravitates towards the use of four household types: single-person, nuclear, extended, and complex. The participant or household informant was an adult female or male aged above 18 years and was responsible for food procurement, preparation, and distribution in the household. The final sample was 280 households who participated in the interviews in their preferred language of Xitsonga, Tshivenda, or English.

### 3.4. Data Collection Procedures

Data were collected from June 2016 to December 2019 using periods of 30 to 60 days at a time. Eleven fieldworkers who were at least bilingual with English competency were trained to conduct the interviews. Ethics approval was granted by the Health and Research Ethics Committee of Stellenbosch University (Ref #: N16/06/083). The ethical principles of voluntary participation, informed consent, anonymity, confidentiality, potential for harm, and results communication were explained to the participants before they agreed to participate. All participants were taken through the purpose of the study, explaining what was required and their right to withdraw at any point of the study, before signing the consent form. The study was conducted according to the Declaration of Helsinki [42] and adhering to the Constitution of South Africa [39]. The household received an incentive of ZAR 375 (South African Rand) (USD 20) for participation, as required by the South African National Health Research Council guidelines [43].

A questionnaire was designed to measure the household demographics profile (household size, gender, income, employment status, social grants received by household members); biophysical environment (type of household, source of water, type of toilet, the energy used for cooking, and household assets or index of wealth); food system inventories, which reflect both household food availability and food access (food purchase places, household food production, food storage, and food procurement); household food preparation and meal distribution; and the food security status of the households.

Household food security was measured using the Household Hunger Scale (HHS) developed by Food and Nutrition Technical Assistance (FANTA) [44]. The HHS is a simple instrument used to measure the level of household food access in the past 30 days in areas of substantial food insecurity. The HHS differs from other household food insecurity indicators in that it has been precisely developed and validated for cross-cultural use [44].

Each of the eight questions of the HHS questionnaire had follow-up sub-questions which were aimed to determine the extent of such food insecurity over 30 days. These questions determine the temporal severity and periodicity of hunger. The households received a score out of eight according to how many “yes” answers were provided. A score of zero (0) indicates food security, a score of 1 to 4 indicates risk of hunger, and a score of 5 to 8 is equal to food insecurity or hunger [44]. These questions were relevant to measuring food security among all household members, including adults and children [44].

The eight questions signify a normally increasing level of severity of food insecurity, and eight “frequency-of-occurrence” questions were asked as a follow-up to every occurrence question to determine how often the condition occurred. The HHS is attached as Appendix A.

Anthropometric measurements, health risk and disease profiles, and dietary patterns, using 24 h recall, a food frequency questionnaire (FFQ), and coping strategy index scale, were also administered. The results are reported elsewhere.

The instruments were reviewed by an academic in the Division of Human Nutrition. In addition, all instruments including measurements were pretested by ten fieldworkers. Data were collected by ten research assistants (dietitians/nutritionists) and ten fieldworkers (Grade 12). The research assistants were trained over five days, while the fieldworkers were trained over two days. The research assistants tested the instruments and procedures on the fieldworkers. The researcher and two other dietitians/academics facilitated the training. Errors were identified and improvements were made. The eleventh research assistant was a coordinator and quality controller and possessed a master’s degree.

Data were collected over three days from each household. Household visit one was carried out to conduct interviews for the demographics profile and biophysical environmental profile of the households (including food availability/inventory) using the main questionnaire, the observation checklist, the HHS questionnaire, and the coping strategies questionnaire. The interview lasted between one and one and a half hours. Household visit two was to measure anthropometric measurements using a record form; conduct an interview using the disease profile and health risk questionnaire and clinical assessment; and recruit participants for blood measurements. The 24 h recall was also carried out on day two. The total time was estimated at two hours for the household, at 30 min per person for measurements. The day-two data were collected from the participants and two children aged under 5, 6 to 12 years, or 13 to 16 years of age. Household visit three for the FFQ was conducted for participants and selected family members. The interview took between one and one and a half hours per person.

### 3.5. Data Analysis

Data were coded, entered, and cleaned using Microsoft Excel 2010 and exported to Statistical Package for the Social Sciences (SPSS) version 26.0. Both descriptive and inferential statistics were gathered. Descriptive data were analyzed using means, standard deviation, and percentages.

A multiple linear regression analysis was performed using the forward stepwise method for three variable blocks, which estimated the effect of the household demographics profile, biophysical environment, and food system factors on household food security scores due to the continuous nature of a dependent variable. The final model estimated the overall effect of the three blocks of variables. The assumption of multicollinearity was verified by using variance inflation factors (VIFs) <10 for all predictors [45]. A *p*-value of less than 0.05 was used as a criterion of statistical significance. Overall, the regression model produced a VIF of less than 10 for all predictors, indicating a non-violation of multicollinearity.

## 4. Results

The demographic characteristics of the households surveyed are shown in Table 1. Out of the 280 households that participated, 279 households were included in the analysis and one household was rejected for incomplete data, giving a 99.6% response rate. The majority of the households had less than four children and/or less than four adults, four females and four males. The average household size was 4.58 ± 2.4 persons (mean and standard deviation), with about half of the households having a family size of fewer than five persons. Regarding the employment status of the members of the households, less than one-third (29.0%) were employed and 65.2% of households had a monthly total income less than ZAR 441 (USD 24). Most of the households (84.6%) received social grants and a few households (5.0%) had a person living with a disability.

The type of house occupied by the households was also assessed—54.5% lived in brick-and-mortar houses (a structural technique in which the bricks are laid out in a systematic pattern and the joints are filled with mortar to make a solid structure), with 27.2% living in hut houses. A greater proportion of the households obtained water from safe sources, namely communal taps (85.7%) and taps inside/outside the house (17.2%). However, about one-third of the households also obtained water from rivers (35.5%) or harvested water from rain (38.8%) and springs/wells (20.8%) as their main sources of water.

Regarding household sanitation, 83.9% used pit latrines and 4.7% used bushes, while 1.5% used flushing toilets located either inside or outside the houses, as shown in Table 2.

Firewood (95.3%) and electricity (36.9%) were the main sources of energy used by the surveyed households for cooking. Data on household assets revealed that more than half had mobile phones (92.1%), televisions (78.9%), radios (57.7%), and cooking stoves, as shown in the table below.

### 4.1. Food Systems Inventory

#### 4.1.1. Food Availability and Affordability at the Village Level

**Figure 2:**
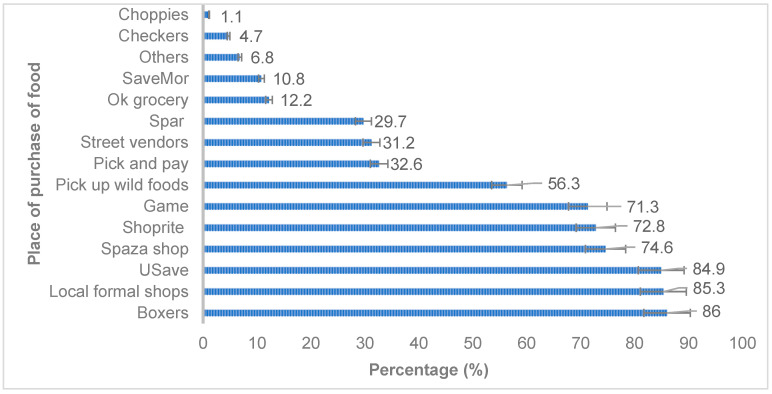
Place of purchase of food by the households by name of supermarkets or stores. Source: Research data.

Data on food availability in the village revealed that households bought their food from more than 14 food shops. More than two-thirds of households bought their food from Boxer supermarkets (86.0%), local formal shops (85.3%), Usave supermarkets (84.9%), Spaza shops (convenience shops, mainly situated in residential areas in South Africa) (74.6%), Shoprite supermarkets (72.8%), and Game retail stores (71.3%), whereas very few households purchased food from another establishment, as depicted in the figure below. The households purchased food from Boxer, Usave, Shoprite, Game, Pick n Pay, Spar, Savemor, Checkers, and Choppies, while one-third bought from street vendors. See Figure 2 below. Boxer, Usave, and Savemor are in Saselamani town, which is located 11 km away from Village One and 5.3 km away from Village Two. Shoprite, Spar, and Pick n Pay are found in Malamulele town, which is located 26.4km away from Village One and 31.5 km away from Village Two. Game and Checkers are located in the bigger towns of Giyani and Thohoyandou, where both villages are 69.2/69.3 km and 52.6/52.7 away, respectively. Street vendors are located in the villages or towns. Households also acquired food in the villages from local shops and spaza shops and picked up wild foods. Furthermore, 83.9% of households bought groceries from food shops every month (27.2%) or more often (19.4%). The modes of transport used by the household to travel to buy food were taxis (89.6%), buses and cars (6.1%), or walking (4.3%). The households used more than one place for food purchases and more than one mode of transportation; thus, the percentages do not add to a hundred.

#### 4.1.2. Food Availability, Storage, and Distribution at the Household Level

Regarding food production, almost all households (91.8%) had backyard fields or home gardens in the yard, while 20.8% had land or fields away from their households (usually at the periphery of the village) used for subsistence farming. The fields are between one and three hectares, since the main goal is to produce food for household consumption. About one-third (31.9%) of the households had an orchard or fruit tree, while 19.4% had a vegetable garden. Few households had a smallholder farm (1.8%) for the production of food for both household consumption and the local markets. The South African Department of Agriculture [45] defined smallholder farmers as those who produce for household consumption and markets, subsequently earning ongoing revenue from their farming businesses, which form a source of income for the family. No household had a large-scale farm for food production for commercial purposes.

In addition, the most domesticated animals for home consumption were chickens (28.3%), cattle (16.8%), goats (8.2%), and pigs (1.4%). The main systems used by the households for food storage were refrigerators (57.3%), deep freezers (49.8%), cupboards (38.0%), and dry rooms (28.3%), as shown in Table 3.

### 4.2. Household Meal Preparation and Distribution

Household food preparation (96.4%) and meal serving or food distribution (96.4%) were mainly undertaken by the adults, although a few households (19.7%) also allowed children to cook and serve food (22.6%). Furthermore, food distribution in the households was undertaken by the following members: everybody (56.3%), the mother/father (26.9%), or the person who cooked (11.8%). An open fire (definition of open fire (*xitiko*): Most common is the three-stone fireplace, although sometimes the stones are ridged clay mounds. Some places instead have metal triangles with legs, or iron bars held by four clay mounds) inside the household (78.9%) and electric stoves (36.2%) were the main resources used for food preparation. See Table 4 below.

### 4.3. Household Food Security

The household food security was assessed using the HHS, and the findings revealed that 36.9% of the households were experiencing hunger, 39.4% were at risk, and 23.7% were food-secure.

The results from the analysis of three regression models are presented in Table 5. Model 1 shows the correlation between the household demographic characteristics, biophysical environment, household food inventory, and household food security score. The number of males in the households, the types of houses occupied by the household members, the source of energy used for cooking, the purchasing of food from street vendors, and the occasional purchasing of food by the households were excluded from Model 1. This model shows that food insecurity was positively influenced by the number of children and adults in the households or the family size, the use of water from communal taps and rivers, and the lack of appropriate food storage facilities. However, receiving child grants and other social grants, the household wealth index (a composite measure of a household's cumulative living standard. The wealth index is calculated using easy-to-collect data on a household’s ownership of selected assets, such as televisions and bicycles; materials used for housing construction; types of water access and sanitation facilities; and ownership of domesticated animals such as cattle), the place of food purchase, and household food production and procurement are not significant factors of food security around the area of study.

Model 2 expanded on model 1 with the addition of more factors, namely the number of males in the household, the type of energy used for cooking, the buying of foods from street vendors, and the occasional purchasing of food. The same variables that were significant in model 1 remain significant in model 2, and none of the added factors influenced the household food security in the study area.

Model 3 elaborated on model 2 by adding the type of house occupied by the household members. Like the previous models, the number of children in the household, large numbers of adults in the households, the use of communal taps and rivers being the main source of water, and inadequate food storage facilities remain significant and positive forces contributing to household food insecurity. However, the effects of all these factors were even stronger when compared to models 1 and 2. They revealed that the five above-mentioned significant factors are the major determinants of food insecurity in the study area. The households’ own food production was an insignificant determinant of food insecurity.

## 5. Discussion

This study aims to understand the influence of household environments, food system inventories, community food environments, and food procurement on household food security. In the literature, we found that the influence of these factors remains ambiguous and contradictory; thus, we seek to report more evidence that could help to provide clarity on the effects of the factors.

### 5.1. Household Demographics

The current study findings revealed that over half of the households had a family size of fewer than five persons, and more than two-thirds of the household adult members were unemployed, with most households living on a monthly total income of less than ZAR 441 (USD 24). Most of the households (84.6%) depend on social grants supported by the government. A social grant in South Africa is given to qualifying poor households to reduce poverty and food insecurity [7], which is consistent with the current study. This study’s findings are also in line with preceding findings, which have also reported that the highest levels of poverty are evident in the rural and peri-urban areas of South Africa due to high levels of unemployment, low monthly total incomes, and high household sizes [46]. In addition, according to the national poverty line set in 2018, a person needed a minimum of ZAR 547 (USD 29) to buy just enough food to meet the nutritional requirements per month in South Africa [47]. A family of four would require ZAR 2188 (USD 116) for food procurement, which is more than four times the total household income observed. The national poverty line in South Africa [47] published by the Statista Research Department on Apr 26 2023 shows that as of 2022, an individual living in South Africa with less than ZAR 945 (USD 54.69) per month was considered poor [47]. This amount is for an individual, not a household, implying that our participants and the households with ZAR 441 (USD 24) were impoverished.

### 5.2. Employment Status

Unemployment was remarkably high at 71% in the study area. According to Statistics South Africa, the unemployment rate was 32.4% in 2019, but the 2022 report revealed that Limpopo’s unemployment rate had risen to 35.6%, a year-on-year increase of 6.2 percentage points from 32.5% in the previous year. Statistics South Africa asserts that unemployment can also negatively affect a household’s food security status [47]. Many studies have shown that when the employment rate is higher, food security is lower, and conversely, when people are employed, they tend to be food-secure [48,49,50]. More recently, a study by Haini et al. [51] examined whether unemployment affects the relationship between income inequality and food security in 143 advanced and developing economies from 2000 to 2019. The authors find that high unemployment rates have a positive relationship with food insecurity in developing countries compared to developed countries, where high levels of unemployment exacerbate the adverse effects of income inequality on food security. When people are employed, they have more financial resources for food procurement. This study showed that 85.6% of households were dependent on government social grants. In South Africa, the government recognizes the significance of uplifting communities and reducing socioeconomic disparities. Social welfare grants are financial assistance programs provided by the South African government to individuals and families who are unemployed and in need of support. These grants aim to alleviate poverty, reduce inequality, and improve the overall well-being of vulnerable populations. Regardless, Kekana, Mbhenyane, and Mabapa reported that the proportion of Child Support Grants spent on food was less than 50%, with most food bought being of low nutritional quality [52]. Waidler and Devereux [53], in their paper to address the question of whether social grants and remittances improve food security and nutritional outcomes, conclude that the proportion of high food expenditure is regarded as 60% and above. Thus, most households that cannot afford to purchase quality and sufficient food are classified as food insecure. This study did not establish the amount of money spent on food by households.

### 5.3. Food Inflation

According to Drimie et al. [54] the prime causes of household food insecurity in South Africa are widespread chronic poverty, rising food prices, and unemployment, which also weaken informal safety nets, especially in urban areas. South Africa has the second largest economy in Africa and has an adequate food supply at the national level, but this is not translated into the food security status of many households [55]. Chakona [56] examined household dietary patterns and food security challenges in peri-urban South Africa and reflected on high unemployment in the wake of rising food prices. The researcher asserted that due to reduced purchasing power, the poor are facing higher food prices but no greater income, have no money to buy food, and they begin to starve and experience food shortages. The National Planning Commission (NPC) of South Africa [57] revealed that a 1% increase in food prices would reduce household welfare by 21.3%. The NPC further estimated that the average income of the poor is less than ZAR 524 (USD 35.70) per month per person in South Africa [57]. A quarter of the population in South Africa lives below the food poverty line of ZAR 561 (USD 38.22) per month [58]. Van Wyk and Dlamini [59] studied the impact of food prices on the welfare of households in South Africa. Their study confirmed a negative correlation between food prices and social welfare. Thus, due to the high unemployment in this current study, price inflation would negatively affect households. The dependency ratio for villages was exceedingly high at 83.3% for Village One, and that of Village Two was 82.8% [40]. This corresponds with the high number of households (84.6%) dependent on grants. The community dependency ratio is a measure of the age structure of a population and relates to the number of individuals who are likely to be economically “dependent” on the support of others. The national total dependency ratio in South Africa is 52.2%, with a youth dependency ratio of 43.8% and an elderly dependency ratio of 8.4% [60]. The implications of a high dependency ratio mean those of working age, and the overall economy, face a greater burden in supporting the dependent population. A report by the World Bank [61] shows that South Africa’s age dependency ratio for the dependent population was 47%, reported in 2019 (latest observation). This is a high value when compared to a global average of 40.1% [61].

The type of house, access to safe water, and sanitary quality at the household level are expected to capture environmental effects that may influence household food security according to Iram and Butt [62]. The 2017 General Household Survey published by Statistics South Africa showed that 62.2% lived in a dwelling/house or brick/concrete block structure on a separate stand, yard, or farm; 8.2% lived in informal dwellings/shacks not in the backyard; 5.5% lived in traditional dwellings/huts/structures made of traditional materials; and 5.4% lived in informal dwellings/shacks in the backyard [63]. This study reveals that 27.2% lived in traditional huts, 54.5% lived in brick-and-mortar houses, and 15.8% lived in shacks. This finding confirms the chronically poor socioeconomic status of the households in the two villages.

A house is an asset; thus, the type of house that a household lives in is a sign of wealth or poverty. The nexus between urbanization and food insecurity in South Africa was examined regarding whether the type of dwelling matters [64]. These researchers adduce that dwelling type is vital in influencing the food insecurity of households. The study further reported that persons living in informal homes are more likely to experience food insecurity than those in semi-formal and formal dwellings. In the current study, some households were living in huts and shacks. In Table 5, model 3 shows the significance of living in a hut or shack and food insecurity in this study. Poor housing infrastructure was found to positively influence food insecurity.

The findings revealed that more than 14 food shops were accessible to the surveyed community, implying that there might be sufficient food availability in the community. These shops were either in the community or in the towns nearby. However, the majority of the households bought food from the food shops in town every month using taxis as the main means of transport. Rideout, Mah, and Minaker [19] asserted that community food environments are measured based on people’s proximity to various kinds of food outlets or the density or variety of diverse types of food outlets within a specific geographic area. Participants from both villages in this study travelled between 5.3 km and 69 km to access big supermarkets in towns. More food purchases took place in the town closer to the villages in Saselamani town, as opposed to Malamulele, Giyani, or Thohoyandou towns.

### 5.4. Own Food Production

Notwithstanding, almost all households (91.8%) were engaged in agriculture and cultivated food mostly for household consumption. However, in this study, the verification of household produce was not undertaken or was an establishment of the last harvest. These findings contradict the results from the national survey that reported that one-third (33.0%) of the households in Limpopo South Africa were engaged in agriculture in 2011 [65] and 21.1% in 2022, according to Census 2022 [66]. The households’ own food production is very encouraging, as it may be targeted for interventions by the government in the form of subsistence farming programs or can be used as a sustainable livelihood approach in communities that still practice it.

### 5.5. Food Insecurity Status

The household food security findings in this study show that less than one-quarter (23.7%) of the surveyed households are food-secure, 39.4% are at risk, and 36.9% are experiencing hunger. This indicates that some households had insufficient food available for the number of people within the house. The findings are not comparable with the South Africa National Health and Nutrition Examination survey, which indicated that 45.6% of the households were food-secure, 28.3% were at risk of hunger, and 26% experienced hunger [55]. Shisana et al. [55] also indicated that most households experiencing hunger are situated in rural formal and urban informal locations [46,55]. Due to the rurality of our study sites, our food insecurity rates are higher in the two villages. Nonetheless, the rate of household food insecurity is low in the present study, contrary to the previous studies conducted in South Africa and Senegal, which reported 64% and 80% [67,68]. This might be due to the use of different food security measurement techniques, different levels of development, and their own food production initiatives. South Africa, with a GDP of USD 368.3B in 2021, was ranked the 35th largest economy in the world, while Senegal was ranked 111th, with USD 24.1B [69]. The current study used the HHS, while later studies used the Household Food Insecurity Access Scale (HFIAS) as a measurement tool. The HHS consists of eight questions and is a simple indicator used to measure household hunger within food-insecure areas. It has been developed and validated for cross-cultural use [70]. The HFIAS is composed of a set of nine questions that have been used in several countries and appear to distinguish food-insecure households from food-secure households across diverse cultural contexts. The HFIAS addresses the shortcomings of the HHS by more clearly capturing problems of both food quality and quantity in the local context. The HFIAS avoids relying heavily on coping strategies that supplement a household’s resource base in assessing food insecurity [71,72]. Comparative information on the various dimensions and determinants of food insecurity in rural Southern Africa’s towns and cities is currently lacking. Most studies focus on urbanization and big cities, as demonstrated by the study on the state of urban food insecurity in Southern Africa [73]. Surprisingly, despite a very high level of self-reported food production and ownership of fields, and projects to support food production by developmental agencies and the government, food insecurity still prevails. This conveys that other determinants require investigation.

### 5.6. Associations between Demographics, Food Environments, and Food Insecurity

The findings from the analysis of the three regression models revealed that the increased number of children and adults or family size in the households, the access and use of water from communal taps and rivers, and limited food storage facilities for food preservation were the major factors that positively influenced the rate of food insecurity in the area of study. The impact of these determinants was even stronger in model 3. Both the number of children and adults in a household have also been reported to positively influence food insecurity in the literature [74]. The probability of being food-insecure increases for households with children and adult members and decreases for households with all elderly members. More household members require more food resources.

Living in a rural area is postulated to increase the probability of being food-insecure, as is a higher unemployment rate [74]. Employment opportunities are not readily available in rural areas due to poor development and lack of industries.

Furthermore, limited water access and use have been reported to be synergistically associated with food availability, access, and utilization [75]. Water is vital for food security; people who have better access to water tend to have lower levels of undernourishment, especially in areas where people depend on local agriculture for food and income [76]. Momberg et al. [77] posit that associations between different forms of malnutrition and environmental conditions, including water, sanitation, and hygiene, may contribute towards persistently poor child health, growth, and cognitive development. The water situation is most critical in sub-Saharan Africa (SSA), where in 2015, 311 million people lacked a safe water source, and more than 70% of SSA populations were living without adequate sanitation [78].

Furthermore, the fetching of water from communal taps and rivers can be a huge time-cost burden, especially for women in rural areas, thus hampering economic and household activities, the earning of extra money, and food preparation in the house. The source of water, whether it is a tap in the house or communal, signals the household wealth status. Poor rural households are likely to have no access to water inside their houses or huts. Ngure et al. [79] reviewed and reaffirmed evidence on the links between clean water, sanitation, and hygiene and stunting and anemia, which are known risk factors for child developmental deficits.

A lack of adequate food storage facilities has been reported to be positively significantly associated with household food security status in concurrence with the current findings, which show that a lack of storage facilities is associated with food insecurity. Another study [80] was aimed at assessing the determinants of household-level food storage, ascertaining consumers’ behavior and perception towards food storage, and assessing the effect of household-level food storage on food safety, wastage, food expenditure, and security. The findings revealed that household-level food storage promotes food safety, reduces food expenditure and waste, and contributes to enhancing food security by 43%. Storage facilities contribute to reducing food losses and offer households the chance to reduce hunger and attain food sufficiency during the lean season [81]. In this study, not all households had cold food-storage facilities, with only 57.3% having refrigerators and 49.8% having deep freezers. Food storage facilities are crucial to improve agricultural incomes and food security for households engaged in smallholder farming [82]. Adeyeye [83] reviewed the role of food processing and appropriate food storage technologies in ensuring food security and availability in Africa. They argue that the use of simple but effective household storage facilities should be promoted to add value to products, ensure food safety, and increase their shelf-life. Wicks, Trevena, and Quine [84] studied the experiences of food insecurity among urban soup kitchen consumers in New Zealand and concluded that a lack of appropriate food storage and cooking facilities were important barriers to adequate nutrition. This study’s findings also confirm that a lack of, limited, or inappropriate food storage facilities is positively associated with food insecurity.

Climate change is intensifying food insecurity across Sub-Saharan Africa, with the Russia–Ukraine war and the COVID-19 pandemic also adding to food shortages and soaring prices. Climate events, which destroy crops and disrupt food transport, are disproportionately common in the Sub-Saharan region, including recent floods in Mozambique, Malawi, Kenya, South Africa, Zambia, Madagascar, Zimbabwe, Tanzania, the Democratic Republic of Congo, Chad, Niger, Côte d’Ivoire, and Nigeria [78]. There are also other disasters like volcanos and conflicts leading to migration and the risk of food insecurity. Lottering, Mafongoya, and Lottering [78], in their review titled “Drought and its impacts on small-scale farmers in sub-Saharan Africa”, contend that the impacts of droughts are far-reaching and affect the environment, societies, and economy of a country. Disasters lead to a lack of clean water and consequently cause infectious diseases like cholera and diarrhea. The Limpopo Province, where this study was conducted, was affected by severe floods in 2000, 2021, and 2023. Nonetheless, the villages in this study, despite being on the Levubu River’s bank, were not washed away.

### 5.7. Limitations

The shortfall of this study includes not using the recent food security measurements used by Statistics South Africa, like the Food Insecurity Experience Scale. A limitation worth noting is that the HHS emphasizes the food quantity measurement of food access and does not measure dietary quality. Also, it does not capture data on food availability, which is a major component of food security. However, the HHS is intended to be used as a more comprehensive food security questionnaire administered to a representative population-based sample of households. The current study complemented the HHS by collecting data and conducting analyses on food availability at the household and community levels. A further limitation was the fact that food production was not quantified in terms of harvest, including seasonality.

In addition, the sample was drawn from similar geographic areas, with the participants having homogenous characteristics. Future research should spread the target set to include semi-urban and urban areas. An in-depth understanding of a household’s food assets and their utilization is important, since many have home vegetable gardens and fruit trees, such as mangoes, avocados, pawpaw, and oranges, but do not see them as a food source. The proportion of household incomes used for food purchasing was not established.

## 6. Conclusions and Policy Implications

The evidence presented in this paper underpins the facts that food insecurity is prevalent among the rural areas of South Africa because of unemployment, which was high (71%) in the studied villages, as well as other variables. Further analysis reveals that an increased household size of children and adults or a large family size, the access and use of water, and limited food storage facilities for food preservation are also major household determinants of food insecurity in the study area. Selepe Mtyingizane and Masuku [85] also revealed that factors that contribute to food insecurity were high levels of unemployment and dependence on state grants in the Mhlontlo Local Municipality, OR Tambo District, Eastern Cape, South Africa. A commendation to the households is the elevated level of food production in homefields or backyard gardens. A high rate of fields for households’ own food production was reported; however, this did not result in any contribution to food availability, as evidenced by the high levels of food insecurity.

A multi-prong and cross-sectoral approach that looks at ways of accelerating the achievement of SDGs 1, 2, 3, and 6, linked to food and nutrition security and other aspirations set in both the local and national spheres of government, should be reinforced, as well as sustainable agriculture production through the promotion of technologies to enhance crops through proper water management and land acquisition. Additionally, alternative solutions, such as the provision of livestock farming to those who have less land, are recommended. The policy implications are that when planning interventions for food insecurity take place, household environmental determinants should be addressed using a multisectoral approach. One strong recommendation that we make is for the government to support rural households that produce their own food by ensuring that they have access to financial resources, land, and water. Regarding the two villages in this study, reinforcement and technology acquisition are required, since they were supported with irrigation systems. Further research should focus on household and community assets and identify pathways for interventions to overcome food shortages.

## Figures and Tables

**Table 1:** Characteristics of households surveyed in the study area (n = 279).

Household Characteristics	Frequency	Percentage
Number of children in the household		
≤3	235	84.2
4–10	44	15.8
Number of adults in the household		
≤3	231	82.8
4–8	48	17.2
Number of females in the household		
≤3	210	75.3
4–13	69	24.7
Number of males in the household		
≤3	241	86.4
4–7	38	13.6
Total household size		
1–4	149	53.4
5–10	123	44.1
11–17	7	2.5
Number of children and teenagers attending school in the household		
Zero	39	14.0
1–3	200	71.7
4–10	40	14.0
Employment status of adults in the household		
Unemployed	198	71.0
Employed	81	29.0
Total household income in ZAR		
0–441 (USD 24)	182	65.2
442–2000 (USD 24–106)	38	13.7
>2000 (USD > 106)	22	7.9
Unknown	37	13.3
Household received social grants		
Yes	236	84.6
No	43	15.4
Household with a person living with a disability		
Yes	14	5.0
No	265	95.0

Source: Research data.

**Table 2:** The biophysical environment of the households in the rural area of South Africa (n = 279).

Type of House	Frequency (n)	Percentage (%)
Brick and mortar	152	54.5
Hut	76	27.2
Shack	44	15.8
Other	7	2.5
**Source of water**		
Communal tap	239	85.7
Tank (harvested from rain)	106	38.0
River	99	35.5
Spring/well	58	20.8
Tap in/outside the house	48	17.2
Borehole	25	9.0
**Type of toilet used by the household**		
Pit latrine	234	83.9
Bush	13	4.7
Flush toilet in/outside the house	4	1.5
**Energy used for cooking in the household**		
Firewood	266	95.3
Electricity	103	36.9
Paraffin and cow dung	2	0.8
**The household assets**		
Mobile phone	257	92.1
Television	220	78.9
Radio	161	57.7
Stove	148	53.0
Video/DVD player	120	43.0
DSTV	82	29.4
Sofas	81	29.0
Microwave oven	46	16.5
Dining table and chairs	43	15.4
Car	22	7.9
Other	14	5.0
House telephone	8	2.9
Play station	5	1.8

DVD: digital versatile disc; DSTV: digital satellite television. Source: Research data.

**Table 3:** Household food production and storage among households in rural South Africa (n = 279).

	Frequency (n)	Percentage (%)
**Household food production**		
Field in the household	256	91.8
Orchard/Fruit tree	89	31.9
Field away the household	58	20.8
Vegetable Garden	54	19.4
Smallholder farm	5	1.8
**Household animal domestication**		
Chickens	79	28.3
Cattle	47	16.8
Goats	23	8.2
Pigs	4	1.4
Other (Sheep, goose turkey, etc.)	11	3.9
**Place of food storage**		
Refrigerator	160	57.3
Deep freezer	139	49.8
Not specific	117	41.9
Cupboard	106	38.0
Dry room storage	79	28.3
Other	12	4.3

Source: Research data.

**Table 4:** Household meal preparation and distribution (n = 279).

Household Meal Preparation and Distribution	Frequency (n)	Percentage (%)
**Household meal preparation**		
Meal preparation by adults	269	96.4
Meal preparation by children	55	19.7
**Resources for food preparation**		
Open fire inside	220	78.9
Electric stove	101	36.2
Open fire outside	55	19.7
Microwave	9	3.2
Other*	12	4.4
**Household food distribution**		
Meal serving undertaken by adults	269	96.4
Meal serving undertaken by children	63	22.6
**Method of food distribution**		
Everybody	157	56.3
The mother/father	75	26.9
The person who cooked	33	11.8

Other* = gas stove, paraffin stove, coal stove, gel stove, and wonder box. Source: Research data.

**Table 5:** The ordinary least squares (OLS) regression analysis for household food security score.

Dependent Variable of Household Food Security	Model 1		Model 2		Model 3	
	Coef.	SE	Coef.	SE	Coef.	SE
**Household sociodemographic characteristics**						
Number children	0.237 **	0.171	0.269 **	0.185	0.268 **	0.186
Number of adults	0.180 **	0.141	0.202 **	0.162	0.200 **	0.163
Number of males			−0.066	0.159	−0.063	0.163
Average child grant	−0.142	0.000	0.000	−0.152	−0.147	0.000
***Ownership of assets***						
Number of assets owned	−0.125	−0.096	−0.122	0.099	−0.118	0.100
**Biophysical environment**						
***Type of house***						
Stayed in a hut (yes, no)					−0.023	0.432
Stayed in a brick-and-mortar house (yes, no)					0.085	0.493
Stayed in a shack (yes, no)					−0.008	−1.254
***Water and sanitation***						
Tap in house	−0.097	1.647	−0.102	1.701	−0.102	1.708
Communal tap	0.201 **	0.524	0.189 **	0.545	0.183 **	0.549
River	0.304 ***	0.415	0.285 ***	0.447	0.306 ***	0.459
Spring/well			0.035	0.455	0.047	0.463
***Energy used for cooking***						
Electricity			0.085	0.612	0.082	0.614
**Food systems inventory**						
***Place of purchase of food***						
Spaza	0.065	0.428	0.055	0.441	0.038	0.449
Boxer	−0.067	0.649	−0.077	0.665	−0.079	0.669
Street vendors			0.019	0.431	0.031	0.438
Pick and pay	−0.054	0.368	−0.046	0.377	−0.046	0.381
USave	−0.065	0.622	−0.062	0.631	0.069	0.637
Pick up wild foods	0.069	0.369	0.055	0.394	0.040	0.402
**Household food production**						
Produced food at home	−0.055	0.804	−0.057	0.834	0.049	0.841
***Ownership of the domesticated animal***						
Ownership of the domesticated animal	0.102	0.437	0.096	0.448	0.076	0.464
***Food storage facilities***						
Refrigerator	−0.064	0.369	0.058	0.379	0.149	1.167
Limited food storage facilities (yes)	0.146 *	1.137	0.142 *	1.157	0.149 *	1.167
**Food preparation**						
Adult food distribution	−0.064	1.059	−0.064	1.073	0.064	1.076
**Food procurement**						
Daily	−0.079	0.385	−0.064	0.426	−0.062	0.429
Weekly	−0.077	0.446	−0.075	0.455	−0.083	0.471
Monthly	−0.088	0.498	−0.091	0.570	−0.094	0.575
Occasionally			−0.002	0.497	−0.008	0.503
More often	0.217	0.389	0.049	0.507	−0.050	0.050
N	279		279		279	
R square	0.466		0.472		0.479	

* *p* < 0.05, ** *p* < 0.001, *** *p* < 0.0001. Coef: coefficients; SE: standard error. Source: Research data.

## Data Availability

Data is available from the corresponding author at Stellenbosch university.

## Abbreviations

FANTA	Food and Nutrition Technical Assistance
FAO	Food and Agriculture Organization
SSA	Sub-Saharan Africa
FFQ	Food Frequency Questionnaire
GDP	Gross Domestic Product
GHI	Global Health Index
HHS	Household Hunger Scale
HFIAS	Household Food Insecurity Access Scale
IFAD	International Fund for Agricultural Development
NDP	South African National Development Plan
SDGs	Sustainable Development Goals
UNICEF	United Nations Children’s Fund
VIF	Variance Inflation Factor
WFP	World Food Program
WHO	World Health Organization
ZAR	South African Rand

## Appendix A. Pink

**Hunger Scale questionnaire**			
**Name of Village:**			**Code:**
**Name of Fieldworker:**			**Code:**
**Name of Household:**			**Code:**
**Date of completion:**			

**Measuring Hunger (Q64–71)**

**HH level insecurity**			**Yes = 1 No = 2**
***Food uncertainty component***	Q64 Does your HH ever run out of money to buy food?	**(a)** In the past 30 days?	
		**(b)** 5 or more days in the past 30 days?	
***Qualitative component***	Q65 Do you ever rely on a limited number of foods to feed your children because you are running out of money to buy food for a meal?	**(a)** In the past 30 days?	
		**(b)** 5 or more days in the past 30 days?	
**Individual level insecurity**			
***Quantitative component***	Q66 Do you ever cut the size of meals or skip because there is not enough money for food?	**(a)** In the past 30 days?	
		**(b)** 5 or more days in the past 30 days?	
	Q67 Do you ever eat less than you should because there is not enough money for food?	**(a)** In the past 30 days?	
		**(b)** 5 or more days in the past 30 days?	
**Child hunger**			
***Quantitative component***	Q68 Do your children ever eat less than you feel they should because there is not enough money for food?	**(a)** In the past 30 days?	
		**(b)** 5 or more days in the past 30 days?	
	Q69 Do your children ever say they are hungry because there is not enough food in the house?	**(a)** In the past 30 days?	
		**(b)** 5 or more days in the past 30 days?	
	Q70 Do you ever cut the size of your children’s meals or do they ever skip meals because there is not enough money to buy food?	**(a)** In the past 30 days?	
		**(b)** 5 or more days in the past 30 days?	
	Q71 Do any of your children ever go to bed hungry because there is not enough money to buy food?	**(a)** In the past 30 days?	
		**(b)** 5 or more days in the past 30 days?	
